# Validation Study of the Spanish Version of the Measure of Happiness (MH) Questionnaire

**DOI:** 10.3390/ejihpe14050090

**Published:** 2024-05-14

**Authors:** Matteo Rizzato, Laura Miraglia, Antonio Francisco Arnau Mollà, Francisco Javier Romero Naranjo, Cinzia Di Dio, Michele Antonelli, Davide Donelli

**Affiliations:** 1 Department of Innovation and Didactic Training, Faculty of Education, Universidad de Alicante, 03690 Alicante, Spain; matteo@matteorizzato.it (M.R.);; 2 Department of Psychology, Università Cattolica del Sacro Cuore, 20123 Milan, Italy; 3 Department of Public Health, AUSL-IRCCS di Reggio Emilia, 42121 Reggio Emilia, Italy; 4 Cardiology Unit, Department of Cardiothoracic and Vascular Diseases, Parma University Hospital, 43126 Parma, Italy

**Keywords:** measure of happiness, Spanish, well-being, happiness, quality of life

## Abstract

This study aims to validate the Spanish version of the Measure of Happiness (MH), a questionnaire that identifies the specific areas of an individual’s life that are related to experienced happiness. The sample consisted of 458 Spanish native speakers (65.7% women, 34.3% men; mean age = 24.14, *SD* = 8.45) and was divided into two groups: Sample 1 (N = 226) underwent Exploratory Factor Analysis (EFA), and Sample 2 (N = 232) underwent Confirmatory Factor Analysis (CFA). The convergent and discriminant validity of the Spanish version of the MH and its subscales was assessed by calculating Pearson correlations between the MH factors and the Subjective Happiness Scale, the State–Trait Anxiety Inventory, the Beck Depression Inventory, and the WHOQOL-BREF. The EFA and CFA confirmed the original five-factor structure of the MH questionnaire. The only difference in the Spanish version of the MH is that Item 4, which was originally assigned to Factor 2 “Financial Status”, was reallocated to Factor 1 “Psychophysical Status”. The reliability of the Spanish version of the MH questionnaire was also confirmed, and the factors correlated in the expected direction with the constructs of quality of life, happiness, anxiety, and depression. The MH questionnaire demonstrated excellent psychometric properties among Spanish respondents.

## 1. Introduction

### 1.1. Background

Happiness is a concept generally describing a positive state of mind and overall well-being in an individual [1]. It differs from transient emotions, such as joy or sadness, by representing a relatively enduring state that encompasses multiple aspects of an individual’s life [2]. The comprehension of happiness is important in philosophy, psychology, academia, and even in different socio-political contexts [3,4,5,6,7,8].

In general, people can often determine whether they are happy or not, but many can find it challenging to articulate the specific reasons behind their happiness. The factors contributing to one’s happiness are diverse, ranging from financial prosperity, levels of activity, job satisfaction, love, spirituality, to family circumstances [9,10]. Even if happiness is primarily regarded as a subjective quality, various endeavours have been made in recent years to objectively assess the degree of one’s subjective happiness through psychometric questionnaires [11,12,13].

Over time, the study of happiness (and its relationship with the individual’s wellbeing) has gained increasing scientific interest. One well-known measure of happiness is Bradburn’s Affect Balance Scale, which evaluates the equilibrium between positive and negative emotions felt within the preceding four weeks, primarily addressing the emotional aspect of happiness [14,15]. Similarly, the Positive and Negative Affect Scale (PANAS) focuses on the emotional facets of well-being, differentiating between positive and negative emotions as separate and distinct concepts [16]. From a cognitive perspective, the Satisfaction With Life Scale (SWLS) [17] and the Delighted–Terrible Scale (DTS) [18] evaluate subjective well-being in terms of life satisfaction. These types of well-being assessments typically ask respondents to rate their emotions over a specific period of time or evaluate their overall quality of life, covering either the emotional or cognitive dimensions of happiness.

The existing questionnaires on happiness and well-being often lack a definite focus on identifying the sources of individual happiness. Additionally, the assessment of happiness and well-being using the aforementioned scales is mainly based on questioning an individual’s emotional and cognitive state in specific time frames or situations. However, none of these measures provide insights into the specific dimensions of a person’s life that are responsible for their overall level of experienced happiness. For these reasons, the Measure of Happiness (MH) was designed: This 14-item questionnaire is constructed with a comprehensive approach and is designed with the goals of being concise, possessing strong psychometric qualities, and being valuable for both research purposes and for the consideration of government bodies, national and international organizations, and social institutions interested in uncovering the determinants of individual or community happiness levels [19]. Following its validation in the Italian language [2], the Measure of Happiness (MH) was planned to undergo a process of translation into other languages. Subsequently, it would be validated through assessments conducted with native speakers hailing from different countries across the globe. This approach aims to ensure the cross-cultural consistency and applicability of the MH, facilitating comparisons across linguistic and cultural groups and providing valuable insights for researchers, policymakers, and organizations interested in understanding the determinants of happiness. These factors comprise psychophysical health, financial stability, the individual’s economic status, personal relationship networks, and future prospects, both on an individual and community scale. The MH questionnaire takes all of these factors into account. Thus, the first step of this project is to validate the MH questionnaire in the Spanish context. The structure of the questionnaire was analysed using an Exploratory Factor Analysis (EFA) to determine the most appropriate factor structure. This was followed by a Confirmatory Factor Analysis (CFA) to confirm the factor structure. The construct validity of the questionnaire was evaluated by correlating it with theoretically related measures. The subsequent sections provide detailed explanations of the methodology and design.

### 1.2. Research Aim

This research aims to evaluate and validate the reliability and validity of the Spanish version of the MH questionnaire among native Spanish speakers.

## 2. Method

### 2.1. Participants

**Figure 1:**
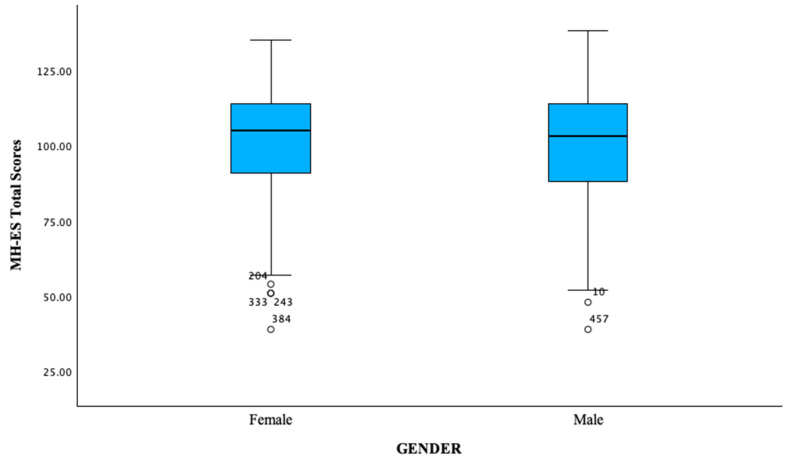
Box plot showing total scores distribution among males and females.

The total sample was composed of four hundred and fifty-eight (458) Spanish native speakers (65.7% women; mean age = 23.21, *SD* = 8.08, range 18–68 years; 34.3% men; mean age = 25.92, *SD* = 8.87, range 18–61 years; age range 19–30: 85.59%, 31–42: 7.21%, 43–65: 6.33%). Of the participants, 44.0% reported having a bachelor’s degree, while 97.2% were Spanish and 72.1% did not use drugs. Table 1 reports the sociodemographic characteristics of the sample. Notably, no participant dropped out of this study, as shown in Figure 1. This study was conducted in accordance with the Declaration of Helsinki and was approved by the Ethics Committee of the University of Alicante, Spain (protocol number: UA-2023-02-20; date of approval: 1 March 2023).

### 2.2. Instruments

**The Measure of Happiness (MH)** [2] assesses happiness by addressing various aspects of an individual’s life. The items were implemented based on events that are most associated with the individual’s well-being. The questionnaire evaluates dimensions of the subject’s everyday life, including their domestic and relational contexts. The original version of the MH was created to reflect the fundamental aspects of the construct. It consisted of 14 items that were grouped into main five factors: Psychophysical Status (F1—3 items), Financial Status (F2—3 items), Relational Private Sphere (F3—3 items), Socio-Relational Sphere (F4—2 items), and Life Perspective (F5—3 items). These scales investigate the subject’s psychophysical health (F1), economic condition (F2), personal relationships (F3 and F4), and long-term goals, life changes, and attitudes toward self-development (F5). Each item is rated on a 10-point Likert scale, ranging from 1 (not at all) to 10 (very, very much). The responses to the 14 items are summed to provide the domains and total scores. The scores for each domain are then added up to calculate the total level of experienced happiness. The English translated version is available in the Supplementary Materials (Supplementary Materials Text S1).

The translation of the MH questions from Italian to Spanish was performed by two native Spanish-speaking academic professionals. Subsequently, another two professionals retranslated the text from Spanish to Italian to verify the translation’s congruence with respect to the original test.

**The Subjective Happiness Scale** (**SHS** [20], Spanish version [21]) is a four-item instrument that measures global subjective happiness on a 1–7 Likert scale. Participants rate themselves or compare themselves to others using statements. The current study’s internal reliability was good (α = 0.78).

**The State–Trait Anxiety Inventory** (**STAI [22,23,24]**, Spanish version [25]) is a 40-item questionnaire divided into two sections: one for assessing state anxiety (Y1, 20 items) and the other for assessing trait anxiety (Y2, 20 items). Respondents answer on a 4-point Likert scale ranging from 1 (not at all) to 4 (very much). State anxiety reflects an individual’s perception of anxiety in a specific moment, such as when taking a test. Trait anxiety, on the other hand, refers to a person’s usual feelings, regardless of their current emotional state. The total score is obtained by summing all scores and being careful to reverse the scores for the positive items. A score above 60 indicates severe anxiety. In the current study, we administered only the STAI which measured the anxiety of the trait, and the internal reliability was good (α = 0.82).

**The Beck Depression Inventory** (**BDI** [26], Spanish version [27]) comprises 21 categories of depressive symptoms, each consisting of four statements. Participants are asked to select one statement that best reflects their level of distress, with scores ranging from 0 (no distress) to 3 (severe distress). The total scale score is calculated by adding up the scores of the 21 items, resulting in a total score that ranges from 0 to 63. In this study, the internal reliability was excellent (α = 0.91).

**The WHOQOL-BREF** is an abbreviated version of the WHOQOL-100 self-report questionnaire ([28]; Spanish version [29]). The WHOQOL-BREF is a questionnaire consisting of 24 items that cover four domains: physical, psychological, social relationships, and environment. Additionally, there are two unscored questions about overall quality of life (QoL) and satisfaction with health. Respondents answer the items on a five-point scale, and domain scores range from 4 to 20, with higher scores indicating a higher QoL. The assessment period for the questionnaire is the past two weeks. In the current study, the internal reliability was excellent (α = 0.90).

### 2.3. Procedure

Data were collected from students and employees of the University of Alicante, Spain, from May 2023 to November 2023. The tests were administered randomly. Each questionnaire was introduced with specific instructions explaining the response criteria and their meanings to the participants. The test concluded after completing the sixth questionnaire. The questionnaires were completed online via a guided procedure. Participants received a standardized invitation message that informed them of this study’s objectives and the experimental nature of participation. Those who decided to participate received a link to access the platform (Survey Monkey) that managed the information and consent before moving on to the next steps. The general objective of this study and the conditions of the procedure were notified to the subjects.

### 2.4. Data Analysis

The sample was divided randomly into two groups. Data from Sample 1 (N = 226; 35.8% male and 64.2% female), with a mean age of 24.22 years (*SD* = 8.36, ranging from 18 to 59 years old), underwent Exploratory Factor Analysis (EFA) to determine the most probable factor structure of the scale. Confirmatory Factor Analysis (CFA) was conducted on data from Sample 2 (N = 232; 32.76% male and 67.24% female) with a mean age of 24.06 years (*SD* = 8.52, ranging from 19 to 68 years old) to evaluate the model fit. The analyses were conducted with SPSS (version 29) and JASP (Version 0.14). To assess the normal distribution of the data, descriptive statistics were computed. Skewness and kurtosis were employed to evaluate the distribution.

Factorial analyses were conducted on two samples to address the issue of common error variance that could arise if the same participants were repeatedly involved in the procedure. The sample’s adequacy was assessed using Bartlett’s test of sphericity (*p* < 0.05) and Kaiser–Meyer–Olkin (KMO > 0.70) test. Hull’s method recommends considering only items with a loading >0.40 on a single factor for further analysis.

To analyse the factor structure of the MH scale, we performed a Principal Component Analysis (PCA) on the data from Sample 1. We used oblique rotation (*promax*) due to the presumed inter-relatedness of factors. Delta was set to 0. We then examined the structure using Cattell’s Scree test technique. We expected a 5-factor model, as was found in the original scale.

We submitted the data from Sample 2 to Confirmatory Factor Analysis (CFA). The fit of the model was assessed by following goodness-of-fit indices: (i) the chi-squared (χ^2^) statistic and its degree of freedom; (ii) the Root Mean Square Error of Approximation (RMSEA) and its 90% confidence interval (90% CI); (iii) the Tucker–Lewis Index (TLI); (4) the Comparative Fit Index (CFI); and (5) the Standardized Root Mean Square Residuals (SRMR). Generally, CFI and TLI values larger than 0.90 are taken to indicate acceptable fit, although values greater than 0.95 are desirable [30]. RMSEA values lower than 0.05 indicate close fit and values between 0.05 and 0.08 indicate acceptable fit [31]. Well-fitting models obtain SRMR values smaller than 0.05 [32].

We tested measurement invariance through Multigroup CFA across gender. Factorial invariance was assessed by comparing a series of models with increasingly strict assumptions about equality across groups [33]. Configural invariance is the basic level of measurement invariance, requiring only the same number of factors and the same overall factor pattern across groups.

Convergent and discriminant validity of the MH-ES and its factor subscales were assessed by computing Pearson correlations between the MH factors and related constructs, i.e., quality of life, happiness, anxiety, and depression.

In order to demonstrate the potential of the questionnaire, the total sample (N = 458) was subjected to two different statistical approaches with nonparametric tests to explore the relationship between the daily use of medicine and happiness levels. First, we conducted a Mann–Whitney U test to compare individuals who use medicine (yes/no) in terms of their self-reported happiness levels. Additionally, we examined the association between the use of medicine and happiness levels using Spearman’s correlation analysis. The results of these analyses were used to inform the discussion section.

## 3. Results

**Figure 2:**
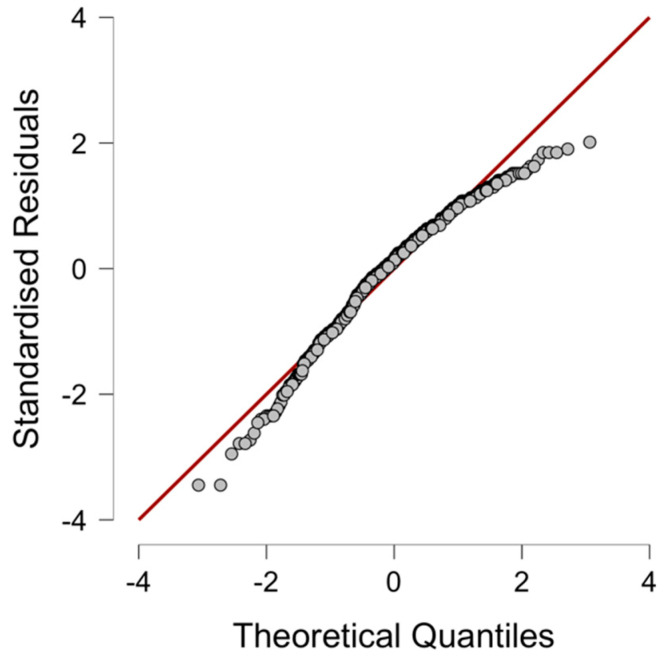
QQ plots showing data skewed to the right.

*Descriptive analysis.* Table 2 summarizes the descriptive statistics for the MH Spanish version. Normal distribution assumption was validated through graphical analysis using normal quantile–quantile (QQ) plots of scores. This plot visually revealed a rightward skew in the data, indicating a departure from the ideal normal distribution (e.g., Figure 2). This right skew suggests that certain scores are higher than expected from a perfectly normal distribution. However, the skewness and kurtosis of the MH-ES total score were between −1 and +1 (−0.70 and 0.30, respectively), and the mean of the total score was 101.94 (*SD* = 18.13).

*Internal consistency.* For all scales, Cronbach’s alpha ranged between 0.72 and 0.90, suggesting moderate and high internal consistency; see Table 2. It suggests that the items in the scale are closely related, and the scale is a reasonably reliable measure of the construct. The removal of any item did not significantly increase Cronbach’s alpha.

**Figure 3:**
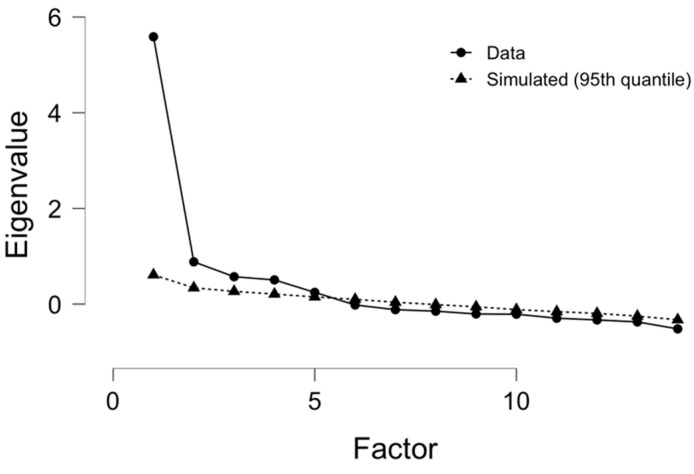
Scree plot.

**Figure 4:**
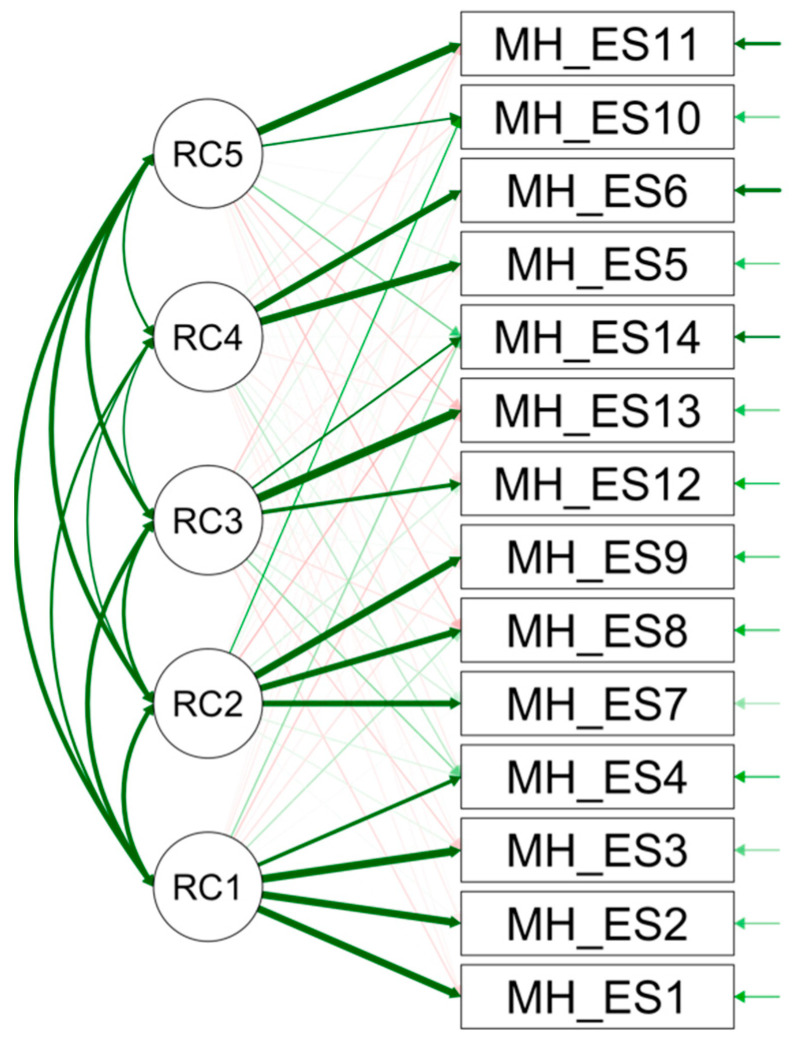
Path diagram.

An Exploratory Factor Analysis was conducted on data from 226 respondents to identify the most likely factor structure of the scale. To explore the suitability of the data for factor analysis, Kaiser–Meyer–Olkin (KMO) and Bartlett’s test of sphericity were performed. The KMO value was greater than 0.60 (KMO = 0.88), and Bartlett’s test of sphericity was statistically significant. Principal Component Analysis was chosen as the method for factor extraction. Promax rotation was used to obliquely rotate the factors. To determine the number of factors, both Kaiser’s criterion (items with eigenvalues greater than 1) [34] and the Scree test [35] were utilized. The criterion of eigenvalues greater than one extracted four factors, which accounted for 72.13% of the total variance. Therefore, a thorough examination of the Scree plot (Figure 3) is a more dependable method for determining the number of factors to be extracted. This is because it takes into account the relative values of the eigenvalues and is not influenced by the number of variables in the analysis [36]. The analysis suggests extracting five factors, which account for 78.44% of the total variance. Factor 1, which is responsible for 46.38% of the variance, includes four items that loaded above 0.65. Factor 2 accounted for 9.64% of the total variance explained, with three items loading above 0.88. Factor 3, with three items loading above 0.60, explained 9.16% of the total variance. Finally, Factor 5 and Factor 4 included two items (with rotated loadings above 0.89 and 0.53, respectively) and accounted for 7.84% and 5.40% of the total variance explained, respectively (see Table 3). EFA results highlighted a specific item, Item 4 (“How fulfilled do you feel with your life at this moment?”), which exhibited notably high factor loadings (rotated loadings of 0.65) on a factor different from the one it was originally. Based on the EFA findings, we made the decision to move Item 4 to *Psychophysics Status* (MH-F1) from its original assignment to *Factor Financial Status* (MH-F2) of the original scale (Figure 4).

**Figure 5:**
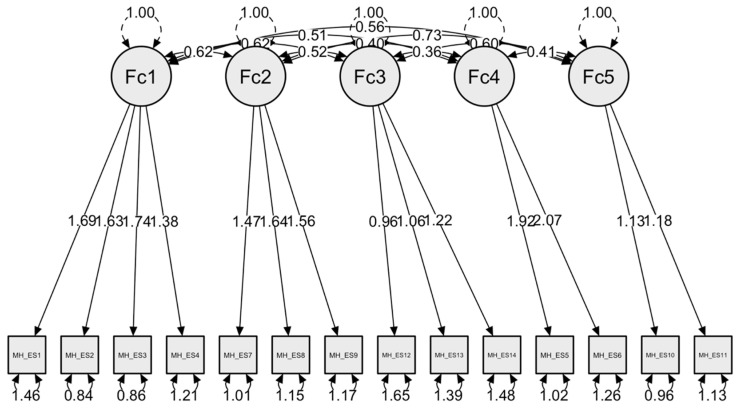
Model plot of the CFA obtained from the 14-item Measure of Happiness Spanish version (MH-ES).

*Confirmatory Factor Analysis (CFA).* The assessment of model fit was based on multiple criteria, including the chi-squared statistic and its degree of freedom, the Root Mean Square Error of Approximation (RMSEA), the Tucker–Lewis Index (TLI) the Comparative Fit Index (CFI), and the Standardized Root Mean Square Residuals (SRMRs). The fit indexes were satisfactory, indicating that the hypothesized factor structure was plausible (see, Table 4): χ^2^/*df* = 2.42, CFI = 0.95, TLI = 0.93, SRMR = 0.04, and RMSEA = 0.08. Importantly, the change in Item 4 underscores the importance of maintaining both statistical rigor and theoretical relevance in the development of our measurement model, aligning it more closely with our research objectives. For a graphical summary of the model, see Figure 5.

*Measurement invariance.* In order to confirm the invariance across gender, a further CFA was carried out (women = 156; men = 76). Although most indices reached the recommended cut-off values, a careful inspection of modification indices (MI) > 10 suggested that correlations between the errors of some pairs of items should be included in the model to improve the model. Multigroup CFA was rerun on the refined and fully unconstrained model and showed adequate fit, χ^2^/*df* = 2.06; CFI = 0.96; TLI = 0.95; SRMR = 0.05; RMSEA = 0.07 (CI = 0.06–0.08), suggesting factorial invariance across gender. Furthermore, we found no deterioration of fit with the constraints of metric and strict invariance, and most indices reached the recommended cut-off values, suggesting that invariance across gender was obtained as shown in Table 5.

*Concurrent validity.* Concurrent validity was assessed by correlating the MH-ES with the Subjective Happiness Scale (SHS) and WHOQOL. Pearson’s correlation between the MH and the SHS was found to be significant. All MH factors showed a significant positive correlation with the SHS, with correlation coefficients ranging between |0.31| and |0.69| (*p* < 0.001), supporting the hypothesized direction. Similarly, all MH-ES factors showed a positive correlation with all WHOQOL factors, with correlation coefficients ranging between |0.32| and |0.82| (*p* < 0.001), indicating high concurrent validity. The correlation statistics are summarized in Table 6.

*Discriminant validity.* Discriminant validity was assessed by comparing scores obtained from the MH-ES with scores obtained from two dispositional constructs that are opposite to that of happiness, namely, anxiety (State–Trait Anxiety Inventory (STAI)) and depression (Beck Depression Inventory (BDI)). The MH factors were found to have a significant negative correlation with both STAI (with *r* ranging between |−0.21| and |−0.50|, *p* < 0.001) and BDI (with *r* ranging between |−0.32| and |−0.65|, *p* < 0.001), indicating a lack of overlap between the constructs. This study found that individuals who scored high on the MH happiness scale also scored low on measures of anxiety and depression. The correlation statistic is presented in Table 6.

## 4. Discussion

The Spanish version of the MH scale exhibited excellent psychometric properties. Both internal consistency and factor analysis confirmed the presence of a five-factor structure in the Spanish MH. A noteworthy finding from the EFA was the reallocation of Item 4, which originally belonged to Factor 2 “Financial Status” in the original scale. The EFA results clearly indicated that this item loaded on a different factor, namely Factor 1 “Psychophysical Status”. The five factors were further validated by Confirmatory Factor Analysis (CFA), thereby confirming consistency with the original structure:

(F1) Psychophysics Status:

1. ¿Cómo valoras la relación con tu cuerpo?
2. ¿Cómo evalúas tu nivel de equilibrio mental y físico?
3. ¿Cómo evalúas tu relación contigo mismo?
4. ¿Cómo de realizado te sientes con tu vida en este momento?

(F2) Financial Status:

5. ¿En qué medida estás satisfecho con su situación económica?
6. ¿En qué medida consideras que su situación financiera es sólida?

(F3) Relational Private Sphere:

7. ¿Cómo valoras la calidad de tus relaciones con tus seres queridos?
8. ¿Hasta qué punto estás satisfecho con el ambiente de tu hogar actual?
9. En tu opinión, ¿en qué medida te valoran los miembros de su familia?

(F4) Socio-Relational Sphere:

10. En tu opinión, ¿hasta qué punto la gente, en general, se siente feliz relacionándose contigo?
11. ¿Cuánto crees que se valoran tus comportamientos en la sociedad?

(F5) Life Perspective:

12. ¿Qué importancia crees que tiene marcarse objetivos a largo plazo?
13. ¿Cómo de interesado estás en tu propia superación personal?
14. ¿Cómo de adaptable te sientes ante los cambios importantes en tu vida?

The alignment of Item 4 with Factor 1 not only underscores its better fit within the domain delineated by this factor but also signifies a robust association between life satisfaction and psychophysical well-being among the Spanish populace. Consistent with this finding, research focussed on the Spanish demographic has consistently shown a positive correlation between healthcare quality and life satisfaction [37]. These findings suggest that individuals in Spain are inclined to intertwine their perception of overall well-being with the state of their health, emphasizing the importance of psychophysical factors in shaping life satisfaction. This correlation assumes particular significance in the aftermath of the COVID-19 pandemic, as individuals have experienced a heightened awareness of their health-related quality of life [38]. In fact, the global health crisis has prompted a collective revaluation of life goals [39]. As a result, the interplay between psychophysical factors and life satisfaction within the Spanish context may reflect the evolving dynamics of societal well-being in the wake of unprecedented global events.

Furthermore, the Spanish MH scale’s five-factor structure demonstrated strong internal consistency, adequate fit across gender, and both convergent and discriminant validity with theoretically related measures. The CFA on the refined and fully unconstrained model indicated satisfactory fit. Additionally, the model demonstrated metric and strict invariance across genders. This result provides further evidence of the MH validity, establishing that it measures the same constructs in men and women and the factor loadings are stable across groups. The questionnaire structure was also supported by the pattern of correlations between the MH-ES and the theoretically correlated measures. The Spanish MH exhibited medium–high positive correlations with SHS and WHOQOL-BREF, thus displaying satisfactory convergent validity. Furthermore, the results demonstrated satisfactory discriminant validity with measures of anxiety (STAI) and depression (BDI), indicating that individuals who scored high on the MH scale had low scores on measures of anxiety and depression.

### 4.1. Potential Applications

**Figure 6:**
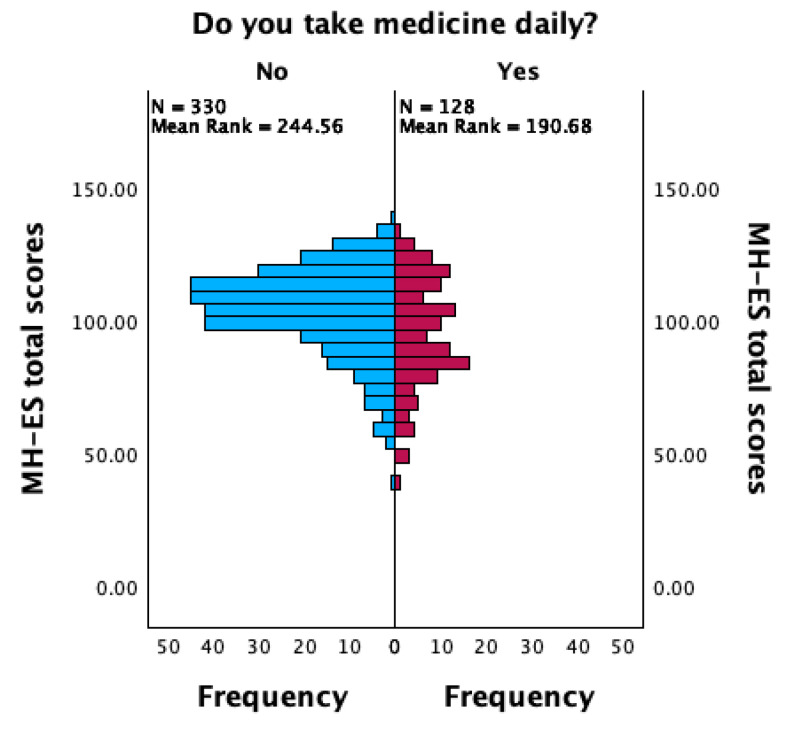
Graphical summary of independent-samples Mann–Whitney U test.

The additional analysis assessed the relationship between the daily use of medicine and happiness levels. The Mann–Whitney U test revealed a statistically significant difference between the two groups (U = 26,089.5, *p* < 0.001), indicating that individuals who take medicines every day tend to have lower happiness levels. The Spearman’s correlation resulted in a negative correlation between the daily use of medicine and happiness levels (*rho* = −0.18, *p* < 0.001). These findings suggest that there is a weak negative association between the use of medicine and happiness levels. Specifically, it indicates that as the daily use of medicine increases, happiness tends to decrease slightly (Figure 6).

The relationship between the daily use of medicine and happiness levels was investigated via nonparametric analyses: the Mann–Whitney U test and Spearman’s correlation. First, the Mann–Whitney U test revealed a significant difference in happiness levels between individuals who use medicine every day and those who do not, indicating that individuals who daily take medicine tend to report lower levels of happiness. Additionally, Spearman’s correlation analysis revealed a weak and negative correlation, suggesting that the extended use of medicine is linked to decreased happiness levels. Assessment of the relationship between diary medicine use and happiness levels provided valuable insights, revealing that individuals who regularly take medication tend to report lower levels of happiness. This underscores the utility of the scale for understanding the emotional well-being of individuals, even in health contexts.

### 4.2. Study Limitations

There are certain limitations to our study. Firstly, even if the sample size was notable, it was, by definition, limited to participants who spoke Spanish. Future studies should concentrate on the cross-cultural comparisons needed to bolster the validity of the MH in cohorts of participants from various backgrounds and countries. Second, although measuring happiness is important, our study only included adult participants. Therefore, in order to collect reliable data in children and adolescents, different scales should be used. Thirdly, although the MH was created as a regular questionnaire, a dynamic tree-like questionnaire could be used in future research as an additional option to consider.

Our study has certain limitations due to the skewed age distribution of our sample, which predominantly comprises younger individuals. This imbalance may introduce biases when assessing older Spanish-speaking individuals, potentially contributing to differences in the structural domains compared to the Italian version. It is important to note that despite demographic differences, the Italian version performed well across various age groups, indicating its robustness and applicability, even among older individuals. Additionally, while factorial invariance was established across gender groups, it was not possible to establish it across different age groups. Conducting future studies with more balanced age distributions would allow higher levels of certainty regarding the underlying structure.

Finally, additional studies should collect more information about the population’s health (diseases, risk factors for life-threatening illnesses, and types of daily medicines) and attempt to investigate which conditions impact happiness the most.

## 5. Conclusions

The main aim of this study was to validate the Spanish version of the MH questionnaire. The Spanish version of the MH showed overall excellent psychometric properties [40]. Both internal consistency and factor analysis confirmed the presence of a five-factor structure in the Spanish MH. The five factors had high internal consistency and moderate-to-strong concurrent and discriminant validity [40]. Both internal consistency and factor analysis supported the reallocation of Item 4, which originally belonged to Factor 2 “Financial Status” in the original scale, into Factor 1 “Psychophysical Status”. The Confirmatory Factor Analysis further confirmed this structure. Additionally, factorial invariance across genders also demonstrated the underlying five-factor structure.

An additional analysis revealed that individuals who regularly take medication tend to report lower levels of happiness (the longer they take daily medical drugs, the more they are likely to rate themselves as unhappy). This underscores the pivotal role of health in overall happiness and the importance of assessing its levels in healthcare settings.

In order to assess how happiness and well-being vary in the general population and which determinants tend to impact their levels the most, future studies should try to measure the levels of happiness in larger and more specific samples of Spanish respondents (for example, elderly people, workers in a specific job sector, and patients with different diseases). The tool could also be used to support policies aimed at making governance choices compatible with the well-being of the population.

## Figures and Tables

**Table 1:** Sociodemographic characteristics.

Sociodemographic Characteristics	Sample *N* = 458
Age, mean ± *SD*	24.14 ± 8.45
Gender	N (%)
Male	157 (65.7%)
Female	301 (34.3%)
Nationality	N (%)
Spanish	445 (97.2%)
Other	13 (2.8%)
Educational level	N (%)
Primary school	5 (1.1%)
Secondary school	3 (0.7%)
High school	181 (39.5%)
Professional training	67 (14.6%)
Bachelor’s degree	202 (44.1%)
Marital status	N (%)
Single	215 (53.1%)
In a relationship	243 (46.9%)
Medicine daily	N (%)
Yes	128 (27.9%)
No	330 (72.1%)

**Table 2:** Range of scores, means, standard deviations, internal consistency, Skewness, and Kurtosis for the Spanish MH (N = 458).

	Range of Scores				Skewness		Kurtosis	
	Min.	Max.	M (*SD*)	α	Statistic	*SE*	Statistic	*SE*
MH-ES	39	138	101.49 (18.13)	0.90	−0.700	0.11	0.297	0.23
MH-ES F1 (Psychophysics Status)	4	40	26.92 (7.08)	0.90	−0.490	0.11	−0.254	0.23
MH-ES F2 (Financial Status)	2	20	12.33 (4.24)	0.86	−0.527	0.11	−0.180	0.23
MH-ES F3 (Relational Private Sphere)	3	30	23.74 (5.34)	0.72	−1.080	0.11	0.908	0.23
MH-ES F4 (Socio-Relational Sphere)	2	20	14.84 (2.98)	0.86	−0.934	0.11	1.312	0.23
MH-ES F5 (Life Perspective)	9	30	23.66 (4.17)	0.72	−0.775	0.11	0.459	0.23

**Table 3:** Pattern matrix presenting loading factors for each item.

	Factor 1 (Psychophysics Status)	Factor 2 (Financial Status)	Factor 3 (Relational Private Sphere)	Factor 4 (Socio-Relational Sphere)	Factor 5 (Life Perspective)
Item 1	0.962				
Item 2	0.865				
Item 3	0.906				
Item 4	0.649				
Item 5				0.889	
Item 6				0.955	
Item 7		0.765			
Item 8		0.908			
Item 9		0.879			
Item 10					0.530
Item 11					1.014
Item 12			0.908		
Item 13			0.899		
Item 14			0.596		
*Extraction Method: Principal Component Analysis.* *Rotation Method: Promax with Kaiser Normalization. ^a^*					

*^a^*. Rotation converged in 6 iterations.

**Table 4:** Values of the goodness-of-fit indices for Confirmatory Factor Analysis.

	Recommended Value	Value Obtained
χ^2^/*df*	≤3.00	2.42
CFI	≥0.90	0.946
TLI	≥0.90	0.926
SRMR	≤0.05	0.044
RMSEA	≤0.08	0.078 ([CI] = 0.063–0.09)

**Table 5:** Goodness-of-fit statistics for testing the multigroup factorial invariance of the Spanish MH across gender.

	Overall Fit Indices				
	χ^2^/*df*	CFI	TLI	SRMR	RMSEA
Recommended value	≤3.00	≥0.90	≥0.90	≤0.05	≤0.08
Configural	1.71	0.95	0.93	0.05	0.07
Metric	1.75	0.95	0.93	0.06	0.08
Strict	2.05	0.90	0.90	0.07	0.09

**Table 6:** Pearson’s correlation analysis between MH-ES factor subscales and the STAI and BDI total scores, the WHOQOL subscales, and SHS mean scores for the sample (N = 232). ** Correlation is significant at the 0.01 level (2-tailed).

	Psychophysics Status (MH-ES F1)	Financial Status (MH-ES F2)	Relational Private Sphere (MH-ES F3)	Socio-Relational Sphere (MH-ES F4)	Life Perspective (MH-ES F5)
SHS	0.69 **	0.31 **	0.50 **	0.58 **	0.53 **
WHOQOL—Environmental	0.54 **	0.35 **	0.33 **	0.32 **	0.47 **
WHOQOL—Physical	0.82 **	0.39 **	0.51 **	0.46 **	0.45 **
WHOQOL—Social relationships	0.47 **	0.33 **	0.53 **	0.45 **	0.33 **
WHOQOL—Psychological	0.59 **	0.54 **	0.53 **	0.52 **	0.43 **
WHOQOL overall	0.60 **	0.43 **	0.45 **	0.40 **	0.38 **
STAI	−0.50 **	−0.21 **	−0.22 **	−0.22 **	−0.25 **
BDI	−0.65 **	−0.35 **	−0.38 **	−0.32 **	−0.39 **

## Data Availability

The data that support the findings of this study are not openly available due to reasons of sensitivity and are available from the corresponding author upon reasonable request. Data are located in controlled access data storage.

## Supplementary Materials

The following supporting information can be downloaded at: https://www.mdpi.com/article/10.3390/ejihpe14050090/s1, Text S1: English version of the MH questionnaire.
