# Supplementary material for: Validation Study of the Spanish Version of the Measure of Happiness (MH) Questionnaire
Source: Eur J Investig Health Psychol Educ. 2024 May 14;14(5):1369–82. doi: 10.3390/ejihpe14050090 (PMC11119619; doi:10.3390/ejihpe14050090)
Supplement: Supplementary file 1 [file ejihpe-14-00090-s001.zip › ejihpe-2879238-supplementary.pdf]

SUPPLEMENTARY MATERIALS

Text S1 English version of the MH questionnaire

The following is the original version of the MH questionnaire (translated from Italian). The different areas are highlighted.

(F1) Psychophysics Status:

1) How do you evaluate your relationship with your body?
2) How do you evaluate your level of mental and physical balance?
3) How do you evaluate your relationship with yourself?

(F2) Financial Status:

4) How fulfilled do you feel with your life at this moment?
5) How satisfied are you with your financial situation?
6) How financially sound do you feel?

(F3) Relational Private Sphere:

7) How do you evaluate the quality of your relationships with your dear ones?
8) At present, how satisfied are you with the atmosphere in your home?
9) In your opinion, how much do your family members appreciate you?

(F4) Socio-Relational Sphere:

10) In general, how happy do you think people are to interact with you?
11) How much do you think your behavior is appreciated in society?

(F5) Life Perspective:

12) How important is it to you to set long-term goals?
13) How much are you engaged in self-improvement?
14) How adaptable do you feel to major changes in your life?
